# Some fixed point theorems in generating space of *b*-quasi-metric family

**DOI:** 10.1186/s40064-016-1867-4

**Published:** 2016-03-03

**Authors:** P. Sumati Kumari, Muhammad Sarwar

**Affiliations:** Department of Mathematics, National Institute of Technology-A.P, Tadepalligudem, Andhra Pradesh India; Department of Mathematics, University of Malakand, Chakdara Dir(L), Khyber PakhtunKhwa Pakistan

**Keywords:** Generating space of b-quasi-metric family, s-h generating b-quasi-contraction, s-z generating b-quasi-contraction, 47H10, 54H25

## Abstract

The purpose of this work is to study some properties of “Generating space of *b*-quasi-metric family”(simply $G_{bq}$-family) and derive some fixed point theorems using some standard contractions. Presented theorems extend and generalize many well-known results in the literature of fixed point theory .

## Background

Banach (1922) established a remarkable fixed point theorem known as the “Banach Contraction Principle.” This renowned principle assures the existence and uniqueness of fixed points of certain self maps of metric spaces and gives a constructive method to find those fixed points.

(**Banach**^1922^) Let (*X*, *d*) be a complete metric space and $f:X\rightarrow X$ be a mapping, there exists a number $t,0\le t<1,$ such that, for each $x,y\in X,$

1 $$\begin{aligned} d(fx,fy)\le td(x,y). \end{aligned}$$

Then *f* has a fixed point.

It is well known that Banach’s contraction principle is one of the decisive result of functional analysis. A huge number of generalizations of the Banach contraction principle have appeared. Of all these, the following generalization of Kannan (1968) and Chatterjea (1972) stands at the top.

(**Kannan**^1968^) Let (*X*, *d*) be a complete metric space and $f:X\rightarrow X$ be a mapping, there exists a number $t,0<t<\frac{1}{2},$ such that, for each $x,y\in X,$

2 $$\begin{aligned} d(fx,fy)\le t[d(x,fx)+d(y,fy)]. \end{aligned}$$

Then *T* has a fixed point.

It is interesting that Kannan’s fixed point theorem is very predominant because Subrahmanyam (1975) proved that, Kannan’s theorem describes the completeness of the metric. In other words, a metric space *X* is complete if and only if every Kannan mapping on *X* has a fixed point.

(**Chatterjea**^1972^) There exists $\alpha \in [0,1)$ such that, for all $x,y\in X,$

3 $$\begin{aligned} d(f(x),f(y))\le \frac{\alpha }{2}[ d(x,f(y))+d(y,f(x))] \end{aligned}$$

Then *f* has a fixed point.

On the other hand, the traditional theory of a metric space has been generalized in wide directions. Some of such generalizations are dislocated metric spaces (Matthews 1986), dislocated quasi-metric spaces (Zeyada et al. 2006), dislocated symmetric spaces Ramabhadra et al. (2014) and quasi-symmetric spaces (Kumari and Ramana 2014) [for more new spaces and related results can be found in Bakhtin (1989), Branciari (2000), Kumari et al. (2012), Kumari et al. (2015), Kumari et al. (2015)].

In 1997, Chang et al. (1997) introduced a definition of “generating space of quasi-metric family” which is a generalization of quasi-metric space. He proved some interesting fixed point theorems and coincidence point theorems in generating space of quasi-metric family.

Later, Lee et al. (1999) define a family of weak quasi-metrics in a generating space of quasi-metric family. He proved Takahashi-type minimization theorem, a generalized Ekeland variational principle and a general Caristi-type fixed point theorem for set-valued maps in complete generating spaces of quasi-metric family by using a family of weak quasi-metrics. He also proved fixed point theorem for set-valued maps in complete generating spaces of quasi-metric family without considering of lower semi-continuity.

Very recently, Kumari and Panthi (2015, 2016) introduced the concepts of “generating space of *b*-dislocated quasi-metric family” (abbreviated “$G_{bdq}$-family”),“generating space of *b*-dislocated metric family” (abbreviated “$G_{bd}$-family”) and “generating space of *b*-quasi-metric family” (abbreviated “$G_{bq}$-family”). Also she proved the existence of unique fixed point theorems in weaker forms of generating spaces by using various cyclic contractive conditions.

Through out this paper, we assume that $$\mathbb {R}^{+}=[0, \infty ),$$$$\mathbb {N}$$ denotes the set of all positive integers .

### **Definition 1**

Let *X* be a non-empty set and $\{d_{\alpha }:\alpha \in (0,1]\}$ a family of mapping $d_{\alpha }$ of $X\times X$ in to $\mathbb {R}^{+}.$ Consider the following conditions for any $x,y,z\in X$ and $s\ge 1.$

$$(d_{1})$$
The family of self distances are zero: $d_{\alpha }(x,x)=0;$
$$(d_{2})$$
The family of distances are symmetric: $d_{\alpha }(x,y)=d_{\alpha }(y,x);$
$$(d_{3})$$
The family of positive distances between distinct points: $d_{\alpha }(x,y)=d_{\alpha }(y,x)=0$ implies $x=y;$
$$(d_{4})$$
For any $\alpha \in (0,1]$ there exist $\beta \in (0,\alpha ]$ such that $d_{\alpha }(x,z)\le s[d_{\beta }(x,y)+d_{\beta }(y,z)];$
$$(d_{5})$$
For any $x,y\in X, \ d_{\alpha }(x,y)$ is non-increasing and left continuous in $\alpha .$

$$d_{(\alpha) }$$ is called,

(1): Generating space of *b*-quasi-metric family if $d_{1}$ through $d_{5}.$
(2): Generating space of *b*-dislocated metric family if $d_{2}$ through $d_{5}.$
(3): Generating space of *b*-dislocated-quasi metric family if $d_{3}$ through $d_{5}.$

If $s=1$ then $G_{bq}$-family becomes generating space of quasi-metric family as defined by Banach (1922).

### *Example 2*

Let (*X*, *d*) be a metric space. If we put $d_{\alpha }$ instead of *d* for all $\alpha \in (0, 1]$ and $x,y\in X$, then $(X, d_{\alpha })$ is a generating space of quasi-metric family.

In Fan (1993), it was proved that each generating space of quasi-metric family generates a topology $\mathfrak {I}_{d_{\alpha }}$ whose base is the family of open balls. The “$G_{bq}$-family” will play a very predominant role in fixed point theory because the class of $G_{bq}$-family is larger than generating space of quasi-metric family.

Motivated by above, In this paper, we establish the existence of a topology induced by a Generating space of *b*-quasi-metric family. Moreover, we derive some unique fixed point theorems.

## Some properties of generating space of *b*-quasi-metric family

In 1880s, A French mathematician H Poincare introduced topological methods in studying nonlinear problems of mathematical analysis. One of main ideas was to utilize fixed point theorems. Together with the study under the topological structure derived from Poincares analysis motivation, L E J Brouwers fixed point theorem came into the world. Since then, the fixed point theory became a major branch of topology and afterwards it consistently became a major theme of the research.

Due to importance of topology in fixed point theory, we discuss some topological structures in *b*-quasi-metric family as below.

### **Definition 3**

Let $$(X,d_{\alpha })$$ be a $$G_{bq}$$-family and $$\{x_{n}\}$$ be a net in *X*. We say that $$\{x_{n}\}$$$$G_{bq}$$-*converges* to *x* in $$(X,d_{\alpha })$$ if $$\lim \nolimits _{n\rightarrow \infty }d_{\alpha }(x_{n},x)=0$$ for all $$\alpha \in (0,1].$$

In this case we write $\lim \nolimits _{n\rightarrow \infty }x_{n}=x.$

### **Definition 4**

Let $(X,d_{\alpha })$ be a $G_{bq}$-family and let $A\subseteq X, \ x\in X.$ We say that *x* is a $G_{bq}$-*limit point* of *A* if there exists a net $\{x_{n}\}$ in $A-\{x_{n}\}$ such that $\lim \nolimits _{n\rightarrow \infty }x_{n}=x.$ The set of all $G_{bq}$-limit points of $A\subseteq X$ is denoted by *D*(*A*).

### **Definition 5**

Let $(X,d_{\alpha })$ be a $G_{bq}$-family with $\epsilon >0, x\in X.$ The set $\mathfrak {B}_{\epsilon }(x)=\{y/d_{\alpha }(x,y)<\epsilon \}$ is called $G_{bq}$-*open ball* of radius $\epsilon$ and center *x*.

The set $\overline{\mathfrak {B}_{\epsilon }}(x)=\{y/d_{\alpha }(x,y)\le \epsilon \}$ is called $G_{bq}$-*closed ball* of radius $\epsilon$ and center *x*.

### *Remark 6*

In a $$G_{bq}$$-family

1. $\lim _{n\rightarrow \infty }x_{n}=x$ if and only if for every $\epsilon >0,$ there exists $n_{0}\in \Delta$ such that $x_{n}\in \mathfrak {B}_{\epsilon }(x)$ for all $n\ge n_{0}.$
2. $$d_{\alpha }(x,y)\le s[d_{\alpha }(x,z)+d_{\alpha }(z,y)]$$ hold.
3. $G_{bq}$-limit point of a net is unique.

Now, we state some propositions and corollaries in $(X,d_{\alpha },\mathfrak {I})$ which can be proved following similar arguments to those given in Kumari (2012), Sarma and Kumari (2012).

### **Proposition 7**

*Let*$$A,B\subseteq X$$*then*

1. $$\mathcal {D}(A)=\phi $$ if $$A=\phi $$
2. $$\mathcal {D}(A)\subseteq \mathcal {D}(B)$$ if $$A\subseteq B$$
3. $$\mathcal {D}(A\cup B)=\mathcal {D}(A)\cup \mathcal {D}(B)$$
4. $$\mathcal {D}(\mathcal {D}(A))\subseteq \mathcal {D}(A)$$

### **Corollary 8**

*If we write*$\overline{A}=A\cup \mathcal {D}(A)$*for*$A\subset X$*the operation*$A\rightarrow \overline{A}$*satisfies Kuratowski’s closure axioms of* Kelley (1960) *so that the set*$\mathfrak {I}=\{A/A\subset X$*and*$\overline{A^{c}}=A^{c}\}$*is a topology onX*.

### **Corollary 9**

*We call*$(X,d_{\alpha },\mathfrak {I})$*the topological space induced by*$G_{bq}$*-family. We call*$A\subset X$*to be closed if*$\overline{A}=A$*and open if*$A\in \mathfrak {I}.$

### **Proposition 10**

*Let*$(X,d_{\alpha })$*be a*$G_{bq}$*-family.*$x\in X$*is a*$G_{bq}$*-limit point of*$A\subset X$*iff for every*$r>0,A\cap \mathfrak {B}_{r}(x)\ne \phi .$

### **Corollary 11**

$x\in \overline{A}$*if and only if*$x\in A$*or*$\mathfrak {B}_{r}(x)\cap A\ne \phi ,$*for all*$r>0.$

### **Corollary 12**

$A\subseteq X$*is open in*$(X,d_{\alpha },\mathfrak {I})$*iff for every*$x\in A,$*there exists*$\delta >0$*such that*$\mathcal {B}_{\delta }(x)\subseteq A.$

### **Proposition 13**

*If*$x\in X$*and*$\delta >0$, *then*$\mathfrak {B}_{\delta }(x)$*is anopen set in*$(X,d_{\alpha },\mathfrak {I}).$

### *Proof*

Let $y\in \mathfrak {B}_{\delta }(x)$ and $0<r<\frac{\delta }{s}-d_{\alpha }(x,y).$ Then $\mathfrak {B}_{r}(y)\subset \mathfrak {B}_{\delta }(x)$

Hence $\mathfrak {B}_{\delta }(x)$ is open. $\square$

### **Proposition 14**

$(X,d_{\alpha },\mathfrak {I})$*is a Hausdorff space.*

### *Proof*

Let $x\ne y$, then $d_{\alpha }(x,y)>0.$ Choose $\delta >0$ such that $2\delta <d_{\alpha }(x,y).$

Then $$\mathfrak {B}_{\frac{\delta }{s}}(x)\cap \mathfrak {B}_{\frac{\delta }{s}}(y)=\phi .$$$$\square $$

### **Corollary 15**

*If*$x\in X,$*then the collection*$\{\mathfrak {B}_{r}(x)/x\in X\}$*isan open base atxin*$(X,d_{\alpha },\mathfrak {I}).$*Hence*$(X,d_{\alpha },\mathfrak {I})$*is first countable.*

*The above corollary yields us to deal with sequences instead of nets.*

### **Definition 16**

A sequence $\{x_{n}\}$ in a $G_{bq}$-family is called a $G_{bq}$-Cauchy sequence if given $\epsilon >0,$ there exists $n_{0}\in \mathbb {N}$ such that for all $n,m\ge n_{0},$ we have $d_{\alpha }(x_{n},x_{m})<\epsilon$ or $\lim \nolimits _{ n\rightarrow \infty } d_{\alpha }(x_{n},x_{m})=0$ for all $\alpha \in (0,1].$

### **Proposition 17**

*Every*$G_{bq}$*-convergent sequence in a*$G_{bq}$*-family is*$G_{bq}$*-Cauchy.*

### **Definition 18**

A $G_{bq}$-family $(X,d_{\alpha })$ is called complete if every $G_{bq}$-Cauchy sequence in *X* is $G_{bq}$-Convergent.

### *Remark 19*

In a $G_{bq}$-family $(X,d_{\alpha }),$ a subset *A* of *X* is said to be closed if for any sequence $\{x_{n}\}$ of points of *A* such that $\lim \nolimits _{ n\rightarrow \infty }x_{n}=x$ then $x\in A.$

## Main results

### **Definition 20**

By $\Phi$ we denote the set of all real functions $\Upsilon :[0,\infty )\rightarrow [0,\infty )$ which have the following properties:

(*a*): $\Upsilon$ is monotone increasing;
(*b*): lim$\Upsilon ^{n}(t)=0$ for any $t>0,$ where $\Upsilon ^{n}(t)=\Upsilon \circ \cdots \circ \Upsilon (t).$

### **Theorem 21**

*Let*$(X,d_{\alpha })$*be a complete*$G_{bq}$*-family with the co-efficient*$s\ge 1, p>1$*and*$T:X\rightarrow X$*satisfy*$s^{p}d_{\alpha }(Tx,Ty)\le \Upsilon (d_{\alpha }(x,y)) \ ; \forall x,y\in X.$*where*$\Phi :\mathbb {R}^{+}\rightarrow \mathbb {R}^{+}$*is a continuous monotone increasing mapping such that*$\lim \nolimits _{n\rightarrow \infty }\Upsilon ^{n}(t)=0$*for each*$t>0.$*ThenThas exactly one fixed point.*

### *Proof*

First of all, note that, $\Upsilon (t)<t$ for all $t>0$ and $\Upsilon (0)<0.$

Let $x_{0}$ be an arbitrary point in *X*. Define the iterative sequence $\{x_{n}\}$ as follows:

$$x_{1}=T(x_{0}),x_{2}=T(x_{1}),\ldots ,x_{n+1}=T(x_{n}),\ldots $$

If we assume that $x_{n+1}=x_{n}$ for some $n\in \mathbb {N}$,then we have $x_{n}=x_{n+1}=T(x_{n}),$ so $x_{n}$ is a fixed point of *T* and the proof is complete. From now on we will assume that for each $n\in \mathbb {N},x_{n+1}\ne x_{n}.$

Consider $$s^{p}d_{\alpha }(x_{n},x_{n+1})= s^{p} d_{\alpha }(Tx_{n-1},Tx_{n}) \le \Upsilon d_{\alpha }(x_{n-1},x_{n})$$

$$d_{\alpha }(x_{n},x_{n+1})\le \frac{1}{s^{p}}\Upsilon (d_{\alpha }(x_{n-1},x_{n})) \le \Upsilon (d_{\alpha }(x_{n-1},x_{n}))$$

By repeating this procedure, we get,

4 $$\begin{aligned} \begin{aligned} d_{\alpha }(x_{n},x_{n+1})&\le \Upsilon d_{\alpha }(x_{n-1},x_{n})\\&\le \Upsilon ^{2} d_{\alpha }(x_{n-2},x_{n-1})\\&\ \ \vdots \\&\le \Upsilon ^{n}d_{\alpha }(x_{0},x_{1}) , \quad\text {for n}>1. \end{aligned} \end{aligned}$$

If $d_{\alpha }(x_{0},x_{1})=0$ then $x_{0}=Tx_{0}$ which yields $x_{0}$ is a fixed point of *T*.

Suppose $$d_{\alpha }(x_{0},x_{1})>0,$$ then $$\lim \nolimits _{n\rightarrow \infty }d_{\alpha }(x_{n},x_{n+1})=0.$$

Which implies for each $\eta \ge 0,$ there exists $k\in \mathbb {N}$ such that

5 $$\begin{aligned} d_{\alpha }(T^{k}(x),T^{k+1}(x))\le \frac{\eta }{2s} \ ; \ \forall k\ge n \end{aligned}$$

Now our aim is to prove $\{x_{n}\}$ is a $G_{bq}$-Cauchy sequence.

If we apply induction with respect to *n*,  to show for all $n\in \mathbb {N},$

6 $$\begin{aligned} d_{\alpha }(T^{k}(x),T^{k+n}(x))\le \frac{\eta }{2s}. \end{aligned}$$

Clearly (3) holds for $n=1.$ Let us assume that (3) holds for some $n\in \mathbb {N}.$

i.e $$d_{\alpha }(T^{k+1}(x),T^{k+n+1}(x))\le \frac{\eta }{2s} $$

We have,

7 $$\begin{aligned} \begin{aligned} d_{\alpha }(T^{k}(x),T^{k+n}(x))&\le \frac{1}{s^{p}}\Upsilon (d_{\alpha }(T^{k}(x),T^{k+n}(x)))\\&\le \frac{1}{s^{p}}\Upsilon (\frac{\eta }{2s})\\&<\frac{\eta }{2s}.\\ \end{aligned} \end{aligned}$$

Now consider,

8 $$\begin{aligned} \begin{aligned} d_{\alpha }(T^{k}(x),T^{k+n+1}(x))&\le s[d_{\alpha }(T^{k}(x),T^{k+1}(x))+d_{\alpha }(T^{k+1}(x),T^{k+n+1}(x))]\\&\le s[\frac{\eta }{2s}+\frac{\eta }{2s}]\\&<\eta .\\ \end{aligned} \end{aligned}$$

Thus by induction, we get, (3) is satisfied for any $n\ge 1.$

Hence $$ d_{\alpha }(T^{k}(x),T^{k+n}(x))<\eta .$$ which yields that $$\{x_{n}\}$$ is a $$G_{bq}$$-Cauchy sequence in a complete $$G_{bq}$$-family. Thus there exists some *u* in *X* such that $$\lim \nolimits _{n\rightarrow \infty }x_{n}=u.$$ Also the subsequence $$\{x_{n+1}\}$$$$G_{bq}$$-converges to *u* in *X*.

For any *x*, *y* in *X*, we have

9 $$\begin{aligned} \begin{aligned} d_{\alpha }(Tx,Ty)&\le \frac{1}{s^{p}}\Upsilon (d_{\alpha }(x,y))\\&<\Upsilon (d_{\alpha }(x,y))\\&\le d_{\alpha }(x,y).\\ \end{aligned} \end{aligned}$$

which implies *T* is continuous. Hence $\lim \nolimits _{n\rightarrow \infty }Tx_{n}=Tu.$

Consider $$d_{\alpha }(u,Tu)\le s[d_{\alpha }(u,x_{n+1})+d_{\alpha }(x_{n+1},Tu)].$$

By taking limits $n\rightarrow \infty , \ d_{\alpha }(u,Tu)=0.$

Hence $$u=Tu.$$

**Uniqueness:** Let *u*, *v* be two fixed points of *T* and $u\ne v.$

Thus $d_{\alpha }(u,v)\ne 0$ which implies $d_{\alpha }(u,v)>0.$

Consider,

10 $$\begin{aligned} \begin{aligned} s^{p}d_{\alpha }(u,v)&=s^{p}d_{\alpha }(Tu,Tv)\\&\le \Upsilon d_{\alpha }(u,v)\\&< d_{\alpha }(u,v).\\ \end{aligned} \end{aligned}$$

A contradiction. Thus $d_{\alpha }(u,v)=0.$ Which implies $u=v.$

Hence *T* has a unique fixed point.

This completes the proof of the theorem. $\square$

By taking $\Upsilon (t)=\lambda t$ with $0\le \lambda <\frac{1}{s^{p}},$ we can set the following corollary which generalizes the famous Banach contraction principle in $G_{bq}$-family.

### **Corollary 22**

*Let*$(X,d_{\alpha })$*be a complete*$G_{bq}$*-family with the coefficient*$s\ge 1,p>1$*and let*$T:X\rightarrow X$*is a mapping such that*$d_{\alpha }(Tx,Ty)\le \lambda d_{\alpha }(x,y)$

*for all*$x,y\in X,$*where*$0\le \lambda <\frac{1}{s^{p}}.$*ThenThas a unique fixed point inX*.

*By taking*$s=1$*in above corollary, we generalize the Banach contraction principle in generating space of quasi-metric family.*

### **Corollary 23**

*Let*$(X,d_{\alpha })$*be a complete generating space of quasi-metric family with the coefficient*$s>1,$*and let*$T:X\rightarrow X$*be a mapping such that*

$d_{\alpha }(Tx,Ty)\le \lambda d_{\alpha }(x,y)$*for all*$x,y\in X,$*where*$0\le \lambda <1.$*ThenThas a unique fixed point inX*.

*By taking*$d_{\alpha }=d$*in above corollary, we get Banach contraction principle in complete metric space.*

Rhoades (1977) *collected some contractive conditions considered by various authors and established implications and non-implications between them. We noted some contractive conditions as mentioned below.*

*Let* (*X*, *d*) *be a metric space. If*$T:X\rightarrow X$*is a self mapping andx*, *ybe any elements ofX*. *Now consider the following contractive conditions:*

(**Rhoades**^1977^) *There exist nonnegative functionsa*, *b*, *csatisfying*

$$\begin{aligned} sup_{x,y\in X}\left\{ a(x,y)+b(x,y)+c(x,y)\right\} \le \lambda <1 \end{aligned}$$

*such that, for each*$x,y\in X,$

$$\begin{aligned} d(Tx,Ty)\le a(x,y)d(x,Ty)+b(x,y)d(y,Tx)+c(x,y)d(x,y). \end{aligned}$$

**Ciric.1** (Ciric 1974) *There exists nonnegative functionsq*, *r*, *s*, *tsatisfying*

$$\begin{aligned} sup_{x,y\in X}\left\{ q(x,y)+r(x,y)+s(x,y)+2t(x,y)\right\} \le \lambda <1 \end{aligned}$$

*such that, for each*$x,y\in X,$

$$\begin{aligned} d(Tx,Ty)\le q(x,y)d(x,y)+r(x,y)d(x,Tx)+s(x,y)d(y,Ty)+t(x,y)[d(x,Ty)+d(y,Tx)]. \end{aligned}$$

**Ciric.2** (Ciric 1971) *There exist a constant*$h, \ 0\le h<1,$*such that for each*$x,y\in X,$

$$\begin{aligned} d(Tx,Ty)\le h.max\left\{ d(x,y),d(x,Tx),d(y,Ty),d(x,Ty),d(y,Tx)\right\} \end{aligned}$$

*Note that above mentioned named contractions, as originally defined by their respective authors. In above contractions, Ciric.2 condition is very significant because, a good number of contractive conditions imply Ciric.2 condition.*

*Based on the definition of quasi-contraction of * Ciric (1971), *we introduce the following definition in the setting of*$G_{bq}$*-family.*

### **Definition 24**

Let $(X,d_{\alpha })$ be a complete $G_{bq}$-family with the parameter $s\ge 1,p>1$. If $T:X\rightarrow X$ be a self mapping which satisfies

11 $$\begin{aligned} s^{p}d_{\alpha }(Tx,Ty)\le h.max\left\{ d_{\alpha }(x,y),d_{\alpha }(x,Tx),d_{\alpha }(y,Ty), d_{\alpha }(x,Ty),d_{\alpha }(y,Tx)\right\} \end{aligned}$$

for all $x,y\in X$ and $h\in [0,\frac{s^{p-1}}{s+1}).$ Then *T* is called “ *s-h generating b-quasi-contraction”.*

### **Definition 25**

Let $(X,d_{\alpha })$ be a complete $G_{bq}$-family with the parameter $s\ge 1,p>1$. If $T:X\rightarrow X$ is a self continuous mapping which satisfies *s-h generating b-quasi-contraction,* then *T* has a unique fixed point in *X*.

### *Proof*

Let $x_{0}$ be an arbitrary point in *X*. Define the iterative sequence $\{x_{n}\}$ as follows:

$$\begin{aligned} x_{1}=T(x_{0}),\;x_{2}=T(x_{1}),\ldots ,x_{n+1}=T(x_{n}),\ldots \end{aligned}$$

If we assume that $x_{n+1}=x_{n}$ for some $n\in \mathbb {N}$,then we have $x_{n}=x_{n+1}=T(x_{n}),$ so $x_{n}$ is a fixed point of *T* and the proof is complete. Now we will assume that for each $n\in \mathbb {N},x_{n+1}\ne x_{n}.$

Now consider,

12 $$\begin{aligned} \begin{aligned} s^{p}d_{\alpha }(x_{n},x_{n+1})&=s^{p} d_{\alpha }(Tx_{n-1},Tx_{n})\\&\le h.max\left\{ d_{\alpha }(x_{n-1},x_{n}),d_{\alpha }(x_{n-1},x_{n}), d_{\alpha }(x_{n},x_{n+1}),d_{\alpha }(x_{n-1},x_{n+1}),d_{\alpha }(x_{n},x_{n})\right\} \\&\le h.max\left\{ d_{\alpha }(x_{n-1},x_{n}),d_{\alpha }(x_{n},x_{n+1}), s\left[ d_{\alpha }(x_{n-1},x_{n})+d_{\alpha }(x_{n},x_{n+1})\right] \right\} \\ \end{aligned} \end{aligned}$$

which implies that,

13 $$\begin{aligned} \begin{aligned} d_{\alpha }(x_{n},x_{n+1})&\le \frac{hs}{s^{p}-hs}d_{\alpha }(x_{n-1},x_{n})\\&\le \frac{h}{s^{p-1}-h}d_{\alpha }(x_{n-1},x_{n})\\&\le c.d_{\alpha }(x_{n-1},x_{n}).\\ \end{aligned} \end{aligned}$$

where $$c=\frac{h}{s^{p-1}-h}$$.

Similarly, by the contractive condition of the theorem, we can get below condition:

14 $$\begin{aligned} d_{\alpha }(x_{n-1},x_{n})\le c.d_{\alpha }(x_{n-2},x_{n-1}) \end{aligned}$$

By repeating the same process, we get for all $n\ge 2.$

15 $$\begin{aligned} \begin{aligned} d_{\alpha }(x_{n},x_{n+1})&\le c.d_{\alpha }(x_{n-1},x_{n})\\&\le \\&\vdots \\&\le c^{n}.d_{\alpha }(x_{0},x_{1})\\ \end{aligned} \end{aligned}$$

Since $0\le c<1$ and applying limits as $n\rightarrow \infty ,$ we get $d_{\alpha }(x_{n},x_{n+1})\rightarrow 0.$

Now our aim is to prove $\{x_{n}\}$ is a $G_{bq}$-Cauchy sequence.

To obtain this, let $m,n>0$ with $m>n.$

Then we have,

16 $$\begin{aligned} \begin{aligned} d_{\alpha }(x_{n},x_{m})&\le s[d_{\alpha }(x_{n},x_{n+1})+d_{\alpha }(x_{n+1},x_{m})]\\&\le sd_{\alpha }(x_{n},x_{n+1})+s^{2}d_{\alpha }(x_{n+1},x_{n+2})+s^{3}d_{\alpha }(x_{n+2},x_{n+3})+\cdots \\&\le sc^{n}d_{\alpha }(x_{0},x_{1})+s^{2}c^{n+1}d_{\alpha }(x_{0},x_{1})+s^{3}c^{n+2}d_{\alpha }(x_{0},x_{1})+\cdots \\&=sc^{n}d_{\alpha }(x_{0},x_{1})[1+sc+(sc)^{2}+(sc)^{3}+\cdots ]\\&\le \frac{sc^{n}}{1-sc}d_{\alpha }(x_{0},x_{1}). \end{aligned} \end{aligned}$$

By taking the limits as $n,m\rightarrow \infty ,$ we get $d_{\alpha }(x_{n},x_{m})\rightarrow 0$ as $cs<1.$

Hence $$\{x_{n}\}$$ is a $$G_{bq}$$-Cauchy sequence in complete $$G_{bq}$$-family $$(X,d_{\alpha }).$$ Thus there exists some $$u\in X$$ such that $$\{x_{n}\}$$$$G_{bq}$$-converges to *u*.

Since *T* is a continuous mapping,

17 $$\begin{aligned} T(u)=T(\lim \limits _{n\rightarrow \infty }x_{n})=\lim \limits _{n\rightarrow \infty }T(x_{n})=\lim \limits _{n\rightarrow \infty }(x_{n+1})=u. \end{aligned}$$

Hence *u* is a fixed point of *T*.

**Uniqueness** Let us suppose that *u* and *v* are two fixed points of *T* where $Tu=u$ and $Tv=v.$ Then by *s-h generating b-quasi-contraction*, we get

18 $$\begin{aligned} \begin{aligned} s^{p}d_{\alpha }(u,v)&= s^{p}[d_{\alpha }(Tu,Tv)]\\&\le h.max \left\{ d_{\alpha }(u,v),d_{\alpha }(u,Tu),d_{\alpha }(v,Tv),d_{\alpha }(u,Tv),d_{\alpha }(v,Tu)\right\} \\&= h.max \left\{ d_{\alpha }(u,v),d_{\alpha }(u,u),d_{\alpha }(v,v),d_{\alpha }(u,v),d_{\alpha }(v,u)\right\} \\&\le h d_{\alpha }(u,v) \end{aligned} \end{aligned}$$

So $d_{\alpha }(u,v)\le k.d_{\alpha }(u,v)$ where $k=\frac{h}{s^{p}}$ and since $0\le k<1,$ then we get $d_{\alpha }(u,v)=0.$

Hence $d_{\alpha }(u,v)=0$ which implies $u=v.$

Hence *T* has a unique fixed point in *X*.

If we take parameter $s=1$ in the above theorem, we obtain following corollary. $\square$

### **Corollary 26**

*Let*$(X,d_{\alpha })$*be a complete generating space of quasi-metric family and*$T:X\rightarrow X$*is a self continuous mapping which satisfies:*

$$\begin{aligned} d_{\alpha }(Tx,Ty)\le h.max\left\{ d_{\alpha }(x,y),d_{\alpha }(x,Tx),d_{\alpha }(y,Ty),d_{\alpha }(x,Ty),d_{\alpha }(y,Tx)\right\} \end{aligned}$$

*for all*$x,y\in X$*and*$h\in [0,\frac{1}{2}).$*ThenThas a unique fixed point inX*.

*If we put*$d_{\alpha }=d$*in above corollary, we get following corollary.*

### **Corollary 27**

*Let* (*X*, *d*) *be a complete metric space and if*$T:X\rightarrow X$*is a self continuous mapping which satisfies:*

$$\begin{aligned} d(Tx,Ty)\le h.max\left\{ d(x,y),d(x,Tx),d(y,Ty),d(x,Ty),d(y,Tx)\right\} \end{aligned}$$

*for all*$x,y\in X$*and*$h\in [0,\frac{1}{2}).$*ThenThas a unique fixed point inX*.

*We now give an example to illustrate the above corollary.*

### *Example 28*

Let $$X=[0,1]$$ and $$d(x,y)=|x-y| \ ; \ \forall x,y\in X.$$

Clearly *d* is a complete metric on *X*. Define the self mapping $T:X\rightarrow X$ by $T(x)=\frac{x}{3}.$

For $x,y\in [0,1],$ we have

19 $$\begin{aligned} \begin{aligned} d(Tx,Ty)&=|\frac{x-y}{3}|\\&=\frac{1}{3}|x-y|\\&=\frac{1}{3}d(x,y)\\&\le h.max\left\{ d(x,y),d(x,Tx),d(y,Ty),d(x,Ty),d(y,Tx)\right\} \end{aligned} \end{aligned}$$

for $\frac{1}{3}\le h<\frac{1}{2}.$ Clearly $x=0$ is the unique fixed point of *T*.

Three eminent conditions (1), (2) and (3) are made significant contribution in the area of fixed point theory and applications. After these three results, a huge number of papers have been written by several authors to those results either improve or generalize some of the conditions (1), (2) or (3), or even the three conditions simultaneously.

In 1972, Zamfirescu (1972) consolidate the (1,2,3) conditions which is known as Zamfirescu contractive condition and proved a fixed point theorem.

**(Zamfirescu**^1972^**)** There exists real numbers $\eta ,\beta ,\gamma , \ 0\le \eta <1, 0\le \beta , \gamma <\frac{1}{2},$ such that for each $x,y\in X$ at least one of the following is true:

1. $$d(Tx,y)\le \eta d(x,y),$$
2. $$d(Tx,Ty)\le \beta [d(x,Tx)+d(y,Ty)], $$
3. $$d(Tx,Ty)\le \gamma [d(x,Ty)+d(y,Tx)].$$

Then *T* has a fixed point.

In Rhoades (1977), Rhoades state below conditions,

(**Rhoades 19**$'$ Rhoades 1977) There exist non-negative functions *a*, *b*, *c* satisfying

$\sup _{x,y\in X}\{a(x,y)+2b(x,y)+2c(x,y)\}\le \lambda <1$ such that, for each $x,y\in X,$

20 $$\begin{aligned} d(f(x),f(y))\le & {} a(x,y)d(x,y)+b(x,y)[d(x,f(x))+d(y,f(y))]\nonumber \\&+c(x,y)\left[ d(x,f(y))+d(y,f(x))\right] \end{aligned}$$

(**Rhoades 19**$''$ Rhoades 1977) There exist a constant $h,\ 0\le h<1,$ such that for each $x,y\in X,$

21 $$\begin{aligned} d(f(x),f(y))\le h\ max\left\{ d(x,y),\ \frac{[d(x,f(x))+d(y,f(y))]}{2},\ \frac{[d(x,f(y))+d(y,f(x))]}{2}\right\} \end{aligned}$$

Moreover, Rhoades proved that Zamfirescus condition is equivalent to Rhoades 19′&Rhoades 19$''$ conditions. However, recently Berinde (2004) proved that Banach’s, Kannan’s, Chatterjea’s and Zamfirescu’s mappings are weak contractions.

### **Theorem 29**

*Let* (*X*, *d*) *is a complete metric space. If*$T:X\rightarrow X$*be a self continuous mapping satisfying any of the conditions either Rhodes or Zamfirescu or Ciric.1. ThenThas a unique fixed point.*

### *Proof*

In Rhoades (1977), Rhodes proved below Implications.

$$\begin{aligned}&Rhodes\Rightarrow Ciric.2\\&\quad Zamfirescu \Rightarrow Ciric.1 \Rightarrow Ciric.2 \end{aligned}$$

Hence from Corollary 27, *T* has a unique fixed point. $\square$

### **Theorem 30**

*Let*$(X,d_{\alpha })$*be a complete*$G_{bq}$*-family with the parameters*$s\ge 1$*and*$p\ge 1$. *If*$T:X\rightarrow X$*is a self continuous mapping which satisfiess-h generating b-quasi-contraction,* i.e.

$$\begin{aligned} s^{p}d_{\alpha }(Tx,Ty)\le h.max\left\{ d_{\alpha }(x,y),d_{\alpha }(x,Tx), d_{\alpha }(y,Ty),d_{\alpha }(x,Ty),d_{\alpha }(y,Tx)\right\} \end{aligned}$$

*for all*$x,y\in X$*and*$h\in [0,\frac{s^{p-1}}{s+1}).$*If for some positive integer*$q, \ T^{q}$*is continuous, thenThas a unique fixed point inX*.

### *Proof*

We can construct a sequence $$\{x_{n}\}$$ as in Theorem 30 and conclude that the sequence $$\{x_{n}\}$$$$G_{bq}$$-converges to some point *u* in *X*.

Thus its subsequence $$\{x_{n_{k}}\}(n_{k}=k)$$$$G_{bq}$$-converges to *u*. Also we have,

$$T^{q}(u)=T^{q}(\lim \limits _{k\rightarrow \infty }x_{n_{k}})=\lim \limits _{k\rightarrow \infty }(T^{q}(x_{n_{k}}))=\lim \limits _{k\rightarrow \infty }(x_{k+q})=u$$

Which yields that *u* is a fixed point of $T^{q}.$

Now we shall prove that $Tu=u.$

Let *l* be a smallest positive integer such that $T^{l}u=u$ but $T^{m}u\ne u. (m=1,2,\ldots l-1).$

If $$l>1$$ then,

22 $$\begin{aligned} \begin{aligned} s^{p}d_{\alpha }(u,Tu)&=s^{p}d_{\alpha }(T^{l}u,Tu)\\&=s^{p}d_{\alpha }(TT^{l-1}u,Tu)\\&\le h.max\left\{ d_{\alpha }(T^{l-1}u,u),d_{\alpha }(T^{l-1}u,T^{l}u),d_{\alpha }(u,Tu),d_{\alpha }(T^{l-1}u,Tu),d_{\alpha }(u,T^{l}u)\right\} \\&\le h.max\left\{ d_{\alpha }(T^{l-1}u,u),d_{\alpha }(u,Tu),s\left[ d_{\alpha }(T^{l-1}u,u)+d_{\alpha }(u,Tu)\right] \right\} \\&\le hsd_{\alpha }(T^{l-1}u,u)+hsd_{\alpha }(u,Tu)\\ \end{aligned} \end{aligned}$$

which yields,

23 $$\begin{aligned} \begin{aligned} d_{\alpha }(u,Tu)&\le \frac{hs}{s^{p}-hs}d_{\alpha }(T^{l-1}u,u)\\&\le \frac{h}{s^{p-1}-h}d_{\alpha }(T^{l-1}u,u).\\ \end{aligned} \end{aligned}$$

Thus $$d_{\alpha }(u,Tu)<k.d_{\alpha }(T^{l-1}u,u)$$; where $$k=\frac{h}{s^{p-1}-h}.$$

Similarly,

24 $$\begin{aligned} \begin{aligned} s^{p}d_{\alpha }(T^{l-1}u,u)&=s^{p}d_{\alpha }(TT^{l-2}u,TT^{l-1}u)\\&\le h.max\left\{ d_{\alpha }(T^{l-2}u,T^{l-1}u),d_{\alpha }(T^{l-2}u,T^{l-1}u),d_{\alpha }(T^{l-1}u,T^{l}u),d_{\alpha }(T^{l-2}u,T^{l}u),d_{\alpha }(T^{l-1}u,T^{l-1}u)\right\} \\&\le h.max\left\{ d_{\alpha }(T^{l-2}u,T^{l-1}u),d_{\alpha }(T^{l-1}u,u),s\left[ d_{\alpha }(T^{l-2}u,T^{l-1}u)+d_{\alpha }(T^{l-1}u,u)\right] \right\} \\&\le hs\left[ d_{\alpha }(T^{l-2}u,T^{l-1}u)+d_{\alpha }(T^{l-1}u,u)\right] \\ \end{aligned} \end{aligned}$$

Which implies,

25 $$\begin{aligned} \begin{aligned} d_{\alpha }(T^{l-1}u,u)&\le \frac{hs}{s^{p}-hs}d_{\alpha }(T^{l-2}u,T^{l-1}u)\\&\le \frac{h}{s^{p-1}-h}d_{\alpha }(T^{l-2}u,T^{l-1}u)\\ \end{aligned} \end{aligned}$$

Inductively we get,

26 $$\begin{aligned} \begin{aligned} d_{\alpha }(T^{l-1}u,u)&=d_{\alpha }(T^{l-1}u,T^{l}u)\\&\le k.d_{\alpha }(T^{l-2}u,T^{l-1}u)\\&\vdots \\&\le k^{l-1}.d_{\alpha }(u,Tu)\\ \end{aligned} \end{aligned}$$

notice that $$k<1.$$

Thus $$d_{\alpha }(u,Tu)\le k^{l-1}.d_{\alpha }(u,Tu)\le d_{\alpha }(u,Tu)$$

Which is a contradiction.

Hence $$Tu=u.$$

Which yields *u* is a fixed point of *T*.

Now if there exists another point $v\ne u$ in *X* such that $Tv=v,$ then

27 $$\begin{aligned} \begin{aligned} s^{p}d_{\alpha }(u,v)&=s^{p}d_{\alpha }(Tu,Tv)\\&\le h.max\left\{ d_{\alpha }(u,v),d_{\alpha }(u,Tu),d_{\alpha }(v,Tv),d_{\alpha }(u,Tv),d_{\alpha }(v,Tu)\right\} \\&\le h.max\left\{ d_{\alpha }(u,v),d_{\alpha }(u,u),d_{\alpha }(v,v),d_{\alpha }(u,v),d_{\alpha }(v,u)\right\} \\&\le hd_{\alpha }(u,v)\\ \end{aligned} \end{aligned}$$

Which implies $d_{\alpha }(u,v)\le \frac{h}{s^{p}}d_{\alpha }(u,v)<d_{\alpha }(u,v).$ A contradiction.

Hence *u* is a unique fixed point of *T* in *X*. $\square$

If we take parameter $s = 1$ in the above theorem, we obtain following corollary in generating spaces of quasi-metric family.

### **Corollary 31**

*Let*$(X,d_{\alpha })$*be a complete generating space of quasi-metric family and*$T:X\rightarrow X$*is a continuous mapping which satisfies:*

$$\begin{aligned} d_{\alpha }(Tx,Ty)\le h.max \left\{ d_{\alpha }(x,y),d_{\alpha }(x,Tx),d_{\alpha }(y,Ty),d_{\alpha }(x,Ty),d_{\alpha }(y,Tx)\right\} \end{aligned}$$

*for all*$x,y\in X$*and*$h\in [0,\frac{1}{2}).$*If for some positive integer*$q, \ T^{q}$*is continuous, thenThas a unique fixed point inX*.

*If we take*$d_{\alpha }=d$*then we get following corollary.*

### **Corollary 32**

*Let* (*X*, *d*) *be a complete metric space and if*$T:X\rightarrow X$*is a continuous mapping which satisfies:*

$$\begin{aligned} d(Tx,Ty)\le h.max\left\{ d(x,y),d(x,Tx),d(y,Ty),d(x,Ty),d(y,Tx)\right\} \end{aligned}$$

*for all*$x,y\in X$*and*$h\in [0,\frac{1}{2}).$*If for some positive integer*$q, \ T^{q}$*is continuous, thenThas a unique fixed point inX*.

Based on the definition of *Zamfirescu Contraction*, we introduce the following definition in the setting of $G_{bq}$-family.

### **Definition 33**

Let $(X,d_{\alpha })$ be a complete $G_{bq}$-family with the parameter $s\ge 1,p\ge 1$. If $f:X\rightarrow X$ be a mapping such that for each $x,y\in X$ at least one of the following is true:

1. $$s^{p}d_{\alpha }(f(x),f(y))\le \eta d_{\alpha }(x,y), \ 0\le \eta <s^{p-1}$$
2. $$s^{p}d_{\alpha }(f(x),f(y))\le \beta [d_{\alpha }(x,f(x))+d_{\alpha }(y,f(y))], \ 0\le \beta <\frac{s^{p}}{s+1}$$
3. $$s^{p}d_{\alpha }(f(x),f(y))\le \gamma [d_{\alpha }(x,f(y))+d_{\alpha }(y,f(x))], \ 0\le \gamma <\frac{s^{p-1}}{s+1}$$

Then *f* is called *“s-z generating b-quasi-contraction”*.

### **Theorem 34**

*Let*$(X,d_{\alpha })$*be a complete*$G_{bq}$*-family with the parameters*$s>1,p>1$. *If*$f:X\rightarrow X$*be a continuouss-z generating b-quasi-contractionthenfhas a unique fixed point inX*.

### *Proof*

Put $y=f(x)$ in the above (1), (2) and (3) of Definition 33, gives,

$$\begin{aligned} d_{\alpha }(f(x),f^{2}(x))\le \frac{\eta }{s^{p}}d_{\alpha }(x,f(x))\\ d_{\alpha }(f(x),f^{2}(x))\le \frac{\beta }{s^{p}-\beta }d_{\alpha }(x,f(x))\\ d_{\alpha }(f(x),f^{2}(x))\le \frac{\gamma s}{s^{p}-\gamma s}d_{\alpha }(x,f(x)) \end{aligned}$$

Choose $h=max\left\{ \frac{\eta }{s^{p}},\frac{\beta }{s^{p}-\beta },\frac{\gamma s}{s^{p}-\gamma s}\right\}$ and $0\le h<\frac{1}{s}$ then we get

28 $$\begin{aligned} d_{\alpha }(f(x),f^{2}(x))\le hd_{\alpha }(x,f(x)) \end{aligned}$$

By repeating this procedure,we obtain

29 $$\begin{aligned} d_{\alpha }(f^{n}(x),f^{n+1}(x))\le h^{n} d_{\alpha }(x,f(x)) \end{aligned}$$

Since $$0\le h<\frac{1}{s},\lim \limits _{n\rightarrow \infty }d_{\alpha }(f^{n}(x),f^{n+1}(x))\rightarrow 0.$$

Now we prove that $f^{n}(x)$ is a $G_{bq}$-Cauchy sequence.

To do this,let *m*, *n* are positive integers such that $m>n.$

Now consider

30 $$\begin{aligned} \begin{aligned} d_{\alpha }(f^{n}(x),f^{m}(x))&\le s[d_{\alpha }(f^{n}(x),f^{n+1}(x))+d_{\alpha }(f^{n+1}(x),f^{m}(x))]\\&\le sd_{\alpha }(f^{n}(x),f^{n+1}(x))+s^{2}d_{\alpha }(f^{n+1}(x),f^{n+2}(x))+s^{3}d_{\alpha }(f^{n+2}(x),f^{n+3}(x))+\cdots \\&\le sh^{n}d_{\alpha }(x,f(x))+s^{2}h^{n+1}d_{\alpha }(x,f(x))+\cdots \\&\le sh^{n}[1+sh+(sh)^{2}+\cdots ]d_{\alpha }(x,f(x))\\&\le \frac{sh^{n}}{1-sh}d_{\alpha }(x,f(x))\\ \end{aligned} \end{aligned}$$

Applying $\lim \limits _{n,m\rightarrow \infty },$ we get, $d_{\alpha }(f^{n}(x),f^{m}(x))\rightarrow 0$ as $hs<1.$

Thus $f^{n}(x)$ is a $G_{bq}$-Cauchy sequence in complete $G_{bq}$-family.

Which implies there exist some $u\in X$ such that $\lim \limits _{n\rightarrow \infty }d_{\alpha }(f^{n}(x),u)=0.$

Since *f* is continuous, we get $f(u)=f(\lim \limits _{n\rightarrow \infty }(f^{n}(x))=\lim \limits _{n\rightarrow \infty }f^{n+1}(x)=u.$

Thus *u* is a fixed point of *f*. Suppose there exists another point $v\ne u$ in *X* such that $f(v)=v$ then $d_{\alpha }(u,v)=d_{\alpha }(f(u),f(v))\le hd_{\alpha }(u,v).$

Since $0\le h<1, \ d_{\alpha }(u,v)=0.$ Which implies $u=v.$ Hence *u* is a unique fixed point.

We now give an example to illustrate the above theorem. $\square$

### *Example 35*

Let $X=[0,1].$ Define $d_{\alpha }:X\times X\rightarrow \mathbb {R}^{+}$ by $d_{\alpha }(x,y)=|x-y|$ for all $\alpha \in (0,1]$ then $d_{\alpha }$ is a complete $G_{bq}$-family. Let $f:X\rightarrow X$ be a mapping defined by $fx=\frac{x}{2}$ then for any $x,y\in X.$

$$\begin{aligned} \frac{|x-y|}{2}<|x-y|<\frac{\eta }{s^{p}}|x-y| \end{aligned}$$

which implies, $$s^{p}\frac{|x-y|}{2}<\eta |x-y|$$

$$i.e. \ s^{p}d_{\alpha }(f(x),f(y))<\eta d_{\alpha }(x,y)$$

Hence *f* satisfies the condition(1) of Theorem 34 but *f* doesn’t satisfy condition(2) of Theorem 34.

Since

$$\begin{aligned} s^{p}d_{\alpha }(f(0),f(1))\nleq \beta [d_{\alpha }(0,f(0))+d_{\alpha }(1,f(1))]. \end{aligned}$$

Hence $x=0$ is the unique fixed point of *f* in *X*.

If we take parameter $s = 1$ in the above theorem, we obtain following corollary in generating spaces of quasi-metric family.

### **Corollary 36**

*Let*$(X,d_{\alpha })$*be a complete generating spaces of quasi-metric family. If*$f:X\times X\rightarrow \mathbb {R}^{+}$*be a continuous mapping such that for each*$x,y\in X$*at least one of the following is true.*

1. $$d_{\alpha }(f(x),f(y))\le \eta d_{\alpha }(x,y), \ 0\le \eta <1$$
2. $$d_{\alpha }(f(x),f(y))\le \beta [d_{\alpha }(x,f(x))+d_{\alpha }(y,f(y))], \ 0\le \beta <\frac{1}{2}$$
3. $$d_{\alpha }(f(x),f(y))\le \gamma [d_{\alpha }(x,f(y))+d_{\alpha }(y,f(x))], \ 0\le \gamma <\frac{1}{2}$$

*Thenfhas a unique fixed point.*

*If we take*$d_{\alpha }=d$*then we get following corollary.*

### **Corollary 37**

*Let* (*X*, *d*) *be a complete metric space. If*$f:X\times X\rightarrow \mathbb {R}^{+}$*be a continuous mapping such that for each*$x,y\in X$*at least one of the following is true.*

1. $$d(f(x),f(y))\le \eta d(x,y), \ 0\le \eta <1$$
2. $$d(f(x),f(y))\le \beta [d(x,f(x))+d(y,f(y))], \ 0\le \beta <\frac{1}{2}$$
3. $$d(f(x),f(y))\le \gamma [d(x,f(y))+d(y,f(x))], \ 0\le \gamma <\frac{1}{2}$$

*Thenfhas a unique fixed point.*

**Open Question** What are the additional conditions as needed in order to establish the existence of a unique fixed point satisfying the condition for any $\alpha \in (0,1]$ there exists $\beta \in (0,\alpha )$ such that $d_{\alpha }(x,z)\le s[d_{\beta }(x,y)+d_{\beta }(y,z)]$ in $G_{bq}$-family $(X,d_{\alpha }).$

**Conclusion** In this work, we introduced a new concept of *s-h generating b-quasi-contraction.*, *s-z generating b-quasi-contraction.*. Also, we derived the existence of fixed point theorems for generating spaces of b-quasi-metric family. Moreover, some examples are provided wherever necessary. Our results may be the motivation to other authors for extending and improving these results to be suitable tools for their applications.
